# Supporting healthful lifestyles during pregnancy: a health coach intervention pilot study

**DOI:** 10.1186/s12884-018-2010-z

**Published:** 2018-09-17

**Authors:** Michael W. Seward, Denise Simon, Martha Richardson, Emily Oken, Matthew W. Gillman, Marie-France Hivert

**Affiliations:** 1 Division of Chronic Disease Research Across the Lifecourse, Department of Population Medicine, Harvard Medical School and Harvard Pilgrim Health Care Institute, Boston, MA USA; 2 Obstetrics & Gynecology, Harvard Vanguard Medical Associates, Boston, MA USA; 3 Environmental Influences on Child Health Outcomes (ECHO) Program, Office of the Director, National Institutes of Health, Bethesda, MD USA; 4 Diabetes Unit, Massachusetts General Hospital, Boston, MA USA

**Keywords:** Gestational weight gain, Goal-setting, Pregnancy, Health coaching

## Abstract

**Background:**

Excessive gestational weight gain (GWG) is associated with adverse health outcomes in both the mother and child. Many previous lifestyle interventions in women with excess weight during pregnancy encouraging appropriate GWG have been unsuccessful, and there remains no consensus about the content, format, or theoretical framework of GWG interventions. We assessed the feasibility and acceptability of a remote health coach intervention to promote healthful lifestyle behaviors and appropriate GWG among overweight pregnant women.

**Methods:**

At one northeastern US clinic, we enrolled 30 overweight (pre-pregnancy BMI **≥** 25 kg/m^2^) pregnant women at a median gestation of 12.5 weeks (IQR: 11–15) into a one-arm trial. We connected participants with a health coach to provide behavioral support to help participants adopt healthful lifestyles during pregnancy. Health coaches contacted participants by phone every 2–3 weeks to monitor goals, and sent emails and text messages between calls. Participants completed baseline (*N* = 30) and follow-up (*N* = 26) surveys at the end of the intervention (36 weeks gestation), as well as follow-up phone interviews (*N* = 18).

**Results:**

Among 30 participants, median age was 32 years (IQR: 28–33), median self-reported pre-pregnancy BMI was 27.3 kg/m^2^ (IQR: 25.7–31.1), and 17/30 were white, 9/30 African-American, and 3/30 Asian. Three-quarters (22/29) of participants completed at least a college degree. Although 25/30 participants reported in baseline surveys that they worried about being able to lose the weight postpartum that they expected to gain during pregnancy, just 12/26 participants reported the same at follow-up (*P* < 0.001). In follow-up surveys, 21/26 participants reported that health coaches were helpful in keeping them motivated, and 22/26 thought the phone conversations helped them face problems and find solutions. Based on qualitative assessment, several themes emerged in follow-up interviews about the quality of the intervention including accountability and support from health coaches. Participants also expressed desire for more visual resources and integration with standard clinical care to improve the intervention.

**Conclusions:**

We demonstrated feasibility and high participant satisfaction with our remote health coach intervention during pregnancy. We identified areas in which we could refine the intervention for inclusion in a full-scale RCT, such as integration with clinical care and additional visual resources.

**Trial Registration:**

Retrospectively registered at ClinicalTrials.gov (NCT03080064, 3/14/2017).

**Electronic supplementary material:**

The online version of this article (10.1186/s12884-018-2010-z) contains supplementary material, which is available to authorized users.

## Background

Pregnancy and post-partum are a key period in the life course for the prevention of obesity and cardiovascular disease in both mothers and children. Over 55% of women of reproductive age in the US are overweight or have obesity [1], and these women are two to three times more likely to experience excessive gestational weight gain (GWG) than women of normal weight [2]. The US Center for Diseases Control (CDC) report on GWG from 2012 to 2013 found that 62% of women with overweight had excessive GWG, compared to 37% of normal weight women [3].

Excessive GWG is associated with several health risks for mothers and their children. Excessive GWG increases the risk of gestational diabetes, cesarean delivery, and post-partum weight retention, which may lead to excess weight later in life [4–7]. Children born to mothers who experienced excessive GWG are more likely to have greater adiposity and other cardiovascular risk factors later in life [8–14]. Interventions that promote healthful lifestyles and limit excessive GWG could therefore possibly help reduce obesity and cardiovascular disease risk in two generations.

Although a few previous behavioral interventions during pregnancy have successfully reduced excessive GWG [15–21], several others have not [22–26]. The two largest RCTs that implemented comprehensive behavioral lifestyle interventions during pregnancy had minimal impact on GWG. The LIMIT study, which included over 2000 women with overweight and obesity, did not find that the intervention impacted GWG [27], and the UPBEAT trial (1500 women with obesity) found only modestly lower GWG (− 0.55 kg) in the intervention group compared to standard care [28]. The reluctance of women to join these trials based on the small proportions of eligible women who decided to participate (19% in UPBEAT, 40% in LIMIT) suggests an additional need to explore the acceptability of behavioral interventions among pregnant women.

Findings from reviews and meta-analyses on the efficacy of GWG interventions are decidedly mixed; even within the few studies that showed some impact of proposed interventions there is no clear consensus about either the content, format, or theoretical framework of GWG interventions [29–33]. Moreover, a review of 5 RCT and 8 qualitative studies before LIMIT and UPBEAT concluded that women’s barriers to behavior change were poorly addressed by existing interventions and that more research is necessary to explore what kinds of interventions are effective. The review found that pregnancy as a period of transition and perceived lack of control emerged as a common theme across the qualitative studies, and suggested that interventions that give women a sense of control may be more effective [30]. This review highlights the need for feasibility studies to ensure proposed interventions are successfully adapted to the needs of pregnant women.

In this mixed methods pilot study, our primary aim was to assess feasibility (recruiting, retention) and acceptability (participant satisfaction) of our intervention. Health coaches used behavioral approaches commonly employed in other behavioral change studies to encourage healthful diets (increase consumption of vegetables and fruits, whole grains, or low mercury fish, decrease fast-food and sugar-sweetened beverage consumption), improve physical activity (increase number of steps/day, increase moderate activity), decrease screen time and optimize sleep duration. We hypothesized that a more flexible delivery of the intervention in terms of the methods of communication with health coaches (e.g. phone call, text, email) would entice recruitment and retention of participants in the study and could promote participant satisfaction with the intervention. We also used surveys and interviews for a secondary goal to explore issues related to weight in pregnancy such as trust in sources of information about weight issues and management, discussions with healthcare providers about weight, and attitudes during pregnancy.

## Methods

### Recruitment

**Fig. 1:**
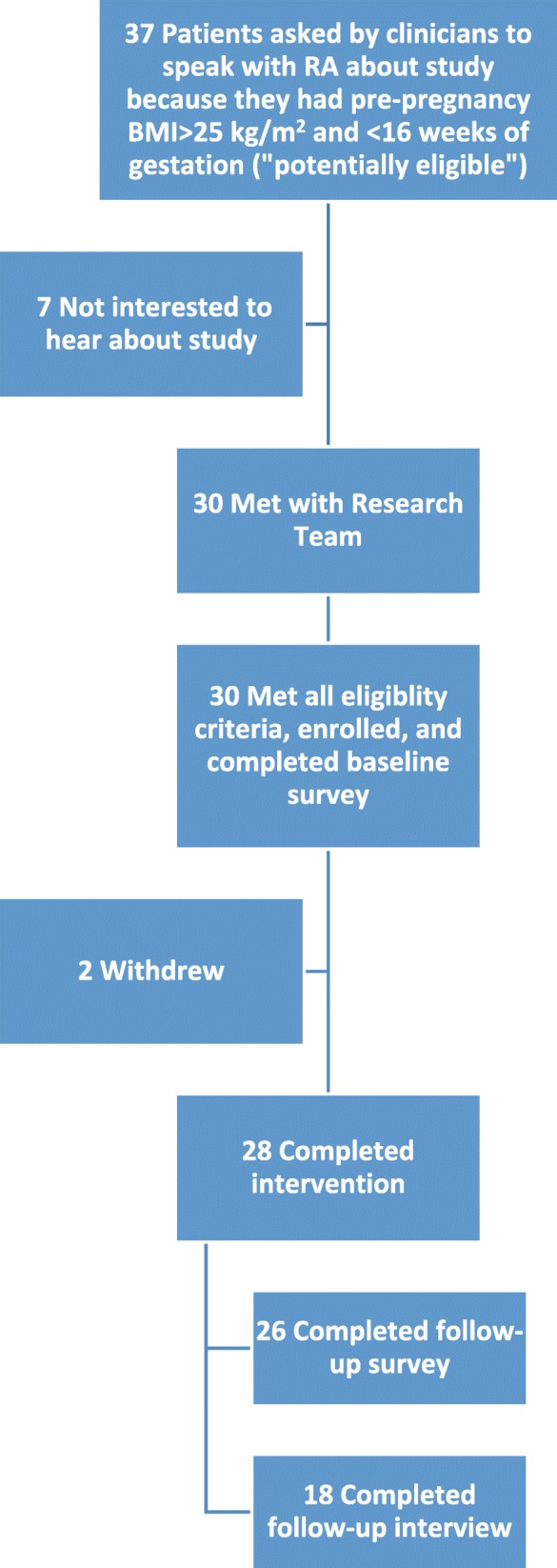
Participant flow chart

We recruited between July 2015 and January 2016 at one northeastern US clinic. We included participants if they were less than 16 weeks’ gestation at the time of their initial obstetric appointment, overweight or had obesity (pre-pregnancy BMI **≥** 25 kg/m^2^), 18 years of age or older, English speaking, and planned to remain at the same obstetric clinic for the duration of their pregnancies. The clinic provided us with monthly lists of appointments for potentially eligible women with pre-pregnancy BMI **≥** 25 kg/m^2^ and less than 16 weeks of gestation. Before each scheduled appointment, we notified healthcare providers that their patients could be eligible for the study. At the end of the clinical encounter, healthcare providers asked patients if they would like to meet with research staff to hear about a healthful lifestyle study. Of 37 individuals approached by the healthcare providers, 30 agreed to meet, and all 30 were eligible and gave written informed consent to participate. Two participants later withdrew from the study during the intervention because of concerns about the time commitment (Fig. 1). Our recruitment goal of 30 women was based on recommendations for conducting qualitative in-depth interviews and the need to account for potential loss to follow-up due to pregnancy events or drop-outs. Because our primary aims of this pilot study were to show feasibility and provide qualitative evaluation of the interventions, we did not attempt to show effect on clinical outcomes for which power calculations would be indicated.

We gave women a $25 gift card after completing baseline surveys at enrollment, and mailed participants another $25 gift card after completing follow-up phone interviews. This study was approved by the Harvard Pilgrim Health Care Human Studies Committee. All participants provided written informed consent at enrollment.

### Study design and sample

Participants completed baseline surveys in person at enrollment, as well as follow-up online surveys and phone interviews at the end of the intervention (36 weeks gestation). Follow-up surveys repeated many of the baseline questions, and additionally asked questions about the intervention. We collected information on demographics, discussions with healthcare providers, attitudes related to weight status and pregnancy, opinions about the intervention, and included general food frequency questions based on surveys used in prior maternal-child health studies conducted at our institution [34]. End of intervention phone interviews followed a guide of open-ended questions to determine insights on what helped achieve goals, motivation, opinions of the health coach intervention, and areas for improvement (Additional file 1: Table S1). A single study staff member (MWS), who had experience with qualitative data collection, conducted all of the individual interviews.

### Health coach intervention

We connected participants at enrollment (median gestation of 12.5 weeks, IQR: 11–15) with a trained health coach who called participants every 2–3 weeks until 36 weeks of gestation. During these phone calls, health coaches helped participants adopt and maintain new healthful lifestyle behaviors that were evidence-based, simple, and easy to track (Additional file 1: Table S2). Goals aimed to promote appropriate gestational weight gain by addressing several behavioral domains including diet, physical activity, screen time, and sleep.

During the first call, health coaches invited participants to prioritize these goals according to their level of self-efficacy, readiness to change, preferences, and values. Specifically, participants were asked to evaluate their lifestyle and identify areas they felt they wanted to focus on to improve their health and wellness. Participants were then asked about barriers to meeting these goals, and this allowed health coaches to create conversations about individual participant’s readiness to make changes and to discuss other resources that may be available to them to establish a prioritization that would be acceptable for each participant. Throughout the intervention, health coaches used principles of motivational interviewing that relied on a patient-centered approach to enhance readiness to change by exploring ambivalence and resistance to change [35]. At each check in, the health coach would discuss goals established and if goals were being met, per the participant report. If the goals were not being met, the health coach would discuss barriers and modify goals to be more realistic to the participants’ individual situation. Health coaches applied several behavioral theories to modify lifestyle including Social Cognitive Theory, Health Belief Model, and Protection Motivation Theory [36–38]. Together, these theoretical bases emphasized the importance of self-regulation, developing specific behavior change plans, monitoring progress towards goals, attaining skills necessary to reach goals, and receiving support through the behavior change process. In addition to setting personal goals adapted to each participant preference and reality, health coaches also presented optimal goals, including targets for ideal cardiovascular health based on the Life’s Simple 7 health factors from the American Heart Association (Additional file 1: Table S2) [39].

During follow-up calls, health coaches monitored progress and helped adjust goals when necessary (e.g. too many goals, or the goal was too ambitious). Health coaches also addressed barriers and potential solutions with participants, and helped them target higher goal settings or select new goals when participants attained them. Health coaches sent emails or text messages depending on participant preferences to check-in about progress toward goals or clinical appointments between calls. Research staff (including an MD) met weekly with health coaches to review their conversations with participants, and to address any medical issues to ensure that it would be reported to the primary care provider obstetric team if appropriate. Although this procedure of notifying the medical team was in place throughout the study, no major concerns were raised to health coaches so there was no direct interaction between the health coaches and the medical team.

We accessed electronic medical records (EMR) and calculated GWG based on pre-pregnancy reported weight and the last weight recorded during pregnancy (> 34 weeks). We defined excessive GWG based on IOM definition per categories (> 11.3 kg of GWG in overweight category, > 9.1 kg of GWG in obesity category).

### Statistical analyses

We measured feasibility via the ease of recruitment based on the ratio of enrolled women to the number of women approached about the study, and via maintenance of contact with a health coach based on the study participant attrition and follow-up survey completion rates (retention). We measured acceptability via survey items about components of the intervention and through qualitative comments from a semi-structured interview.

### Surveys

We performed all analyses using SAS software version 9.4 (SAS Institute Inc., Cary, NC). For most questions, participants chose between four categories: strongly agree, somewhat agree, somewhat disagree, and strongly disagree. For the group of questions asking about attitudes related to weight status and pregnancy, we pooled answers into “agree” and “disagree” for simplicity (Table 3). We conducted McNemar-Bowker symmetry tests to examine differences between baseline and follow-up survey responses.

### Interviews

We transcribed phone interviews verbatim during the calls, and completed content analysis of the transcripts using principles of the immersion-crystallization method [40]. This qualitative technique includes multiple rounds of “immersion” through close readings of transcripts, followed by reflection and the “crystallization” of emerging themes. Three investigators (MWS, DS, and MFH) read the transcripts, and one investigator (MWS) coded the transcripts. The three investigators (MWS, DS, and MFH) had to arrive at a unanimous consensus concerning the emerging themes after reading the verbatim transcripts. We reported the 5 themes that emerged from the analysis with representative quotes.

## Results

### Participant characteristics

Participants at baseline (*N* = 30) had a median age of 31.5 years (IQR: 28.3–33), a median self-reported pre-pregnancy BMI of 27.3 kg/m^2^ (IQR: 25.7–31.1), and 57% (17 of 30) were white, 30% (9 of 30) African-American, and 10% (3 of 30) Asian. Three-quarters (75%, 22 of 29) of participants completed a college degree or higher level of education. Two-thirds (67%, 20 of 30) of participants were married, 23% (7 of 30) were single and living with a partner or a significant other, and 10% (3 of 30) were single. Thirteen percent (4 of 30) reported an annual household income of $40,000 or less, 20% (6 of 30) reported incomes between $40,001 and $100,000, and 60% (18 of 30) reported incomes above $100,000 (Table 1).

**Table 1:** Characteristics of participating women at baseline

Characteristics	Median (IQR) or N (%)
	*N* = 30
Age, years	31.5 (28.3–33.0)
Gestational age at enrollment, weeks	12.5 (11.0–15.0)
Self-reported pre-pregnancy BMI, kg/m^2^	27.3 (25.7–31.1)
Primiparous	23 (77)
Race/Ethnicity	
White	17 (57)
Black or African American	9 (30)
Asian	3 (10)
Other	1 (3)
Highest level of education	
High school graduate	2 (7)
Some college	5 (17)
College graduate	12 (41)
Graduate school	10 (34)
Marital Status	
Single	3 (10)
Single & living with partner or significant other	7 (23)
Married	20 (67)
Household Income ($)	
$40,000 or less	4 (13)
$40,001 - $100,000	6 (20)
$100,001 - $150,000	10 (33)
More than $150,000	8 (27)
Don’t know	2 (7)

### Feasibility

Most (81%, 30 of 37) of the women potentially eligible to join the study and 100% (30 out of 30) of women approached by research staff about the study enrolled as participants. Participants completed baseline (*N* = 30) surveys in person at enrollment, as well as follow-up online surveys (*N* = 26) and phone interviews (*N* = 18) at the end of the intervention (36 weeks gestation) (Fig. 1).

### Surveys

#### Trust in information sources about weight-related issues in pregnancy

Large proportions of participants at baseline reported “a lot of trust” in advice related to weight gain during pregnancy given by doctors (93%, 28 of 30), midwives (92%, 24 of 26), and prenatal or childbirth classes (65%, 13 of 20). Fewer participants said they put a lot of trust in husbands or partners (29%, 6 of 21), friends or family (20%, 4 of 20), and pregnancy books or magazines (8%, 2 of 24). No participants reported a lot of trust in advice from the internet or television (Table 2). We saw similar trends at follow-up: women put a lot of trust in doctors (88%, 21 of 24), midwives (81%, 17 of 21), and prenatal or childbirth classes (73%, 8 of 11). Fewer participants put a lot of trust in pregnancy books or magazines (26%, 5 of 19), the internet (18%, 3 of 17), husbands or partners (15%, 2 of 13), or friends and family (13%, 2 of 16). No participants reported a lot of trust in advice from the television.

**Table 2:** Level of trust for various sources of advice related to weight gain during pregnancy reported by participants at baseline

Source	N*	Do not trust at all N (%)	Trust a little N (%)	Trust a lot N (%)
Doctor	30	0 (0)	2 (7)	28 (93)
Midwife	26	0 (0)	2 (8)	24 (92)
Husband/partner	21	2 (10)	13 (62)	6 (29)
Friends and family	20	4 (20)	12 (60)	4 (20)
Pregnancy books or magazines	24	2 (8)	20 (83)	2 (8)
Internet	24	6 (25)	18 (75)	0 (0)
Television	19	11 (58)	8 (42)	0 (0)
Prenatal or childbirth classes	20	1 (5)	6 (30)	13 (65)

*Number of participants who answered each question

#### Discussions with healthcare providers about weight and health behaviors during pregnancy

At baseline, half of the participants (50%, 15 of 30) reported that healthcare providers (doctors and/or midwives) had discussed the risks of gaining too much weight during pregnancy, almost all women (97%, 28 of 29) reported healthcare providers had discussed diet, most (80%, 24 of 30) reported healthcare provider discussions concerning physical activity, and half (50%, 15 of 30) concerning sleep. At follow-up, more participants (81%, 21 of 26) reported that their healthcare providers had discussed the risks of gaining too much weight, almost all women (96%, 25 of 26) reported healthcare provider discussions about diet, the vast majority (88%, 23 of 26) about physical activity, and over three-quarters (77%, 20 of 26) about sleep.

#### Attitudes towards weight-related issues in pregnancy

We found substantial differences between baseline and follow-up surveys for two of the attitudes queried (Table 3). While over three-quarters (77%, 23 of 30) of participants reported in baseline surveys that they worried they may get fat during pregnancy, fewer participants (58%, 15 of 26) reported the same at the end of the intervention (*P* = 0.01). Similarly, although most (83%, 25 of 30) said at baseline that they worried about being able in post-partum to lose the weight that they would gain during pregnancy, under half (46%, 12 of 26) reported the same at follow-up (*P* < 0.001) (Table 3).

**Table 3:** Attitudes related to weight status and pregnancy reported by participants at baseline (median gestation of 12.5 weeks) and at the end of the intervention (median gestation of 36.0 weeks)

Opinions or Worries	Baseline		End of intervention		*P*-value**
	N*	Agree, N (%)	N*	Agree, N (%)	
I am proud of looking pregnant	30	25 (83)	26	23 (88)	0.65
I think a pregnant woman is beautiful	30	29 (97)	26	26 (100)	1.00
I like my maternity clothes	29	13 (45)	26	15 (58)	0.48
I worry that I may get fat during this pregnancy	30	23 (77)	26	15 (58)	0.01
The weight that I’ve gained during this pregnancy makes me feel unattractive	29	10 (34)	25	9 (36)	1.00
I am embarrassed at how big I have gotten during this pregnancy	28	6 (21)	26	3 (12)	0.32
As long as I’m eating a well-balanced diet, I don’t care how much I gain	30	9 (30)	26	14 (54)	0.06
If I gain too much weight one month, I try to keep from gaining the next month	30	8 (27)	26	5 (19)	0.53
I tried to keep my weight down so I didn’t look pregnant earlier on	30	6 (20)	26	3 (12)	0.18
Just before going to the doctor, I try not to eat	30	2 (7)	26	3 (12)	0.56
I worry that I will have a difficult time losing the weight I’ve gained during this pregnancy	30	25 (83)	26	12 (46)	0.0009

^*^Number of participants who answered each question

^**^P-value calculated from the difference between baseline and end of intervention surveys using the McNemar test

Among attitudes that we did not find to have meaningful differences between baseline and follow-up, the vast majority in baseline (83%, 25 of 30) and follow-up (88%, 23 of 26) surveys said they were proud of looking pregnant. All 26 women thought a pregnant woman is beautiful. However, over a third (36%, 9 of 25) said the weight they gained during pregnancy makes them feel unattractive, and only a few (12%, 3 of 26) said they were embarrassed at how big they got during pregnancy. At the end of pregnancy, over half (54%, 14 of 26) said they did not care how much weight they gain as long as they eat a well-balanced diet (Table 3).

#### Health coach intervention acceptability and satisfaction

In follow-up surveys, most participants (81%, 21 of 26) reported that the health coach was helpful in selecting and setting goals, and that the health coach was helpful in keeping them motivated. Over three-quarters (77%, 20 of 26) thought the health coach was helpful in measuring and monitoring lifestyle goals. The large majority (85%, 22 of 26) said phone conversations helped them face problems and find solutions, while slightly fewer (70%, 16 of 23) said personalized text messages or emails were helpful reminders (Table 4).

**Table 4:** Opinions about the health coach intervention reported by participants at the end of intervention

	N*	Strongly disagree N (%)	Somewhat disagree N (%)	Somewhat agree N (%)	Strongly agree N (%)
The health coach was helpful in selecting and setting goals for myself.	26	1 (4)	4 (15)	12 (46)	9 (35)
The health coach was helpful to keep me motivated.	26	1 (4)	4 (15)	14 (54)	7 (27)
The health coach was helpful to measure and monitor my lifestyle goals.	26	1 (4)	5 (19)	14 (54)	6 (23)
The phone conversations help me to face problems and find solutions.	26	1 (4)	3 (12)	15 (58)	7 (27)
The personalized text messages or emails were helpful reminders.	23	3 (13)	4 (17)	10 (43)	6 (26)

^*^Number of participants who answered each question

Participants most commonly selected goals to increase vegetable and fruit intake (77%, 20 of 26), increase physical activity (50%, 13 of 26), and increase the number of steps per day (42%, 11 of 26). In self-evaluations of the level of achievement for selected goals, participants did best with dietary goals: the majority fully achieved an increase in vegetables and fruit (60%, 12 of 20), an increase in whole grains (57%, 4 of 7), and a decrease in fast-food (56%, 5 of 9). Participants had the least success with physical activity and sleep goals: about a quarter fully achieved an increase in the number of steps per day (27%, 3 of 11) and more optimal sleep (25%, 2 of 8) (Table 5).

**Table 5:** Goals selected during the intervention and self-evaluation on the level of achievement for each selected goal by the participants at the end of intervention

Domain	Goal	Number of participants selecting each goal	Did not achieve N (%)	Somewhat achieved N (%)	Fully achieved N (%)
Diet	Increase vegetables and fruits intake	20	0 (0)	8 (40)	12 (60)
Diet	Increase whole grains intake	7	0 (0)	3 (43)	4 (57)
Diet	Decrease fast-food intake	9	0 (0)	4 (44)	5 (56)
Diet	Decrease sugar-sweetened beverages	6	0 (0)	3 (50)	3 (50)
Diet	Increase low-mercury fish intake	5	0 (0)	3 (60)	2 (40)
Physical Activity	Increase physical activity	13	4 (31)	4 (31)	5 (38)
Screen time	Decrease screen time	6	1 (17)	3 (50)	2 (33)
Physical Activity	Increase number of steps/day	11	2 (18)	6 (55)	3 (27)
Sleep	Optimize sleep duration	8	1 (13)	5 (63)	2 (25)
	Other	3	0 (0)	0 (0)	3 (100)

Despite self-reported success achieving dietary goals, we did not find differences between self-reported baseline and follow-up consumption of food, beverages, or fast-food in the overall group (Additional file 1: Table S3-S5). Although we did not measure GWG, we did access EMR weight data and found that 10/28 women who completed the intervention had excessive GWG, as defined by the IOM.

### Post-intervention interviews

We conducted 18 individual follow-up phone interviews for a mean of 12 min each (range: 8–20). Several important themes emerged about the quality of the intervention including motivation for personal health and the health of the baby, as well as accountability and support from health coaches. Participants also suggested ways to improve the intervention such as integration with standard clinical care and expressed desire for more visual resources such as mobile apps and tracking tools (Table 6).

**Table 6:** Representative quotes for emerging themes from 18 follow-up phone interviews

Theme	Representative Quote
Motivation	• “I want to be able **to get my body back** once I have the baby.” • “I didn’t want **high blood pressure**, and thankfully I don’t have it anymore.” • “Making sure the **baby** is healthy…number one was ‘what does he need?’”
Accountability	• “It was nice…to have someone that made me **accountable**…I didn’t have to do it on my own.” • “It was really good to have a **3rd party** outside of family and friends... [The health coach] helped me set goals and helped me keep track of where I stood with those goals.” • “It was good having someone there that was sort of **checking in on you** so that you had that **urge to please someone**”
Support	• “I had **someone on my team**…so it was good positive reinforcement. It was great to bounce stuff off...My coach helped me be able to better grasp the need to do these things and I think at this point, the fruit has become **a habit that I will keep after the baby.** Now I’m loving the fruit. He comes in with tangerines and now I’m tearing the fruit up!” • **“**She was great and **supportive.”** • **“**I always thought **she was like a friend and she would go above and beyond to find answers...I felt like I could go to her** even if it wasn’t for a call. If had a question I could reach out to her.”
Wishes for more Integration	• “…if it was more closely tied with the doctor’s appointments…It was **too removed from the medical** side.” • “more **integration with the doctors** in general. If you’re already going in to see the doc once a month it would be great to see someone [a health coach] there already.” • “If the health coach had **access to medical records** she could ask what has happened over the last two weeks if she sees you gained 2 pounds. Sometimes you don’t want to admit you gained weight to health coach, so it would be better if she already knew.”
Desire for More Resources	• “There’s no way to track your goal other than a conversation, so if there was some **study app** that made it a little more automated.” • “I want **more resources**, not just a checkin to talk.” • “Maybe offering some **guidelines in the beginning**…starting off with ‘try these meal planners’ or ‘try this calorie tracking app.’” • “All the goals are just discussed verbally. Maybe having more concrete goals that are maybe a **worksheet** or something.”

Boldface text highlights representative quotes from themes that emerged across all phone interviews

### Motivation

The majority (11 of 18) cited primarily their own personal health as motivation to achieve lifestyle goals. Most of these women listed “general wellbeing” or “staying healthy and fit” as important drivers. Many participants also mentioned their bodies as motivation: “I want to be able to get my body back once I have the baby,” or “I definitely did not want to come out of pregnancy sloppy.” One woman said she “didn’t want high blood pressure,” and another said she is a “happier person when I’m moving and active.”

A few (3 of 18) cited principally the health of the baby as motivation: “I just want to have a healthy baby.” Another woman stated: “Making sure the baby is healthy to be honest with you. It was my number one motivation. I don’t want to deprive him of anything, taking prenatal vitamins every day, and yeah number one was ‘what does he need?’” Others (4 of 18) mentioned both personal and maternal sources of motivation: “I wanted to stay healthy for me and the baby.”

### Accountability

Several women discussed how the health coach intervention made them take responsibility for their lifestyle goals: “It was nice…to have someone that made me accountable…I didn’t have to do it on my own.” One woman particularly liked how the health coach filled a unique role as an independent source of feedback and described how “it was really good to have a third party outside of family and friends... [The health coach] helped me set goals and helped me keep track of where I stood with those goals.” Most women agreed that the health coaches provided helpful check-ins: “It was good having someone there that was sort of checking in on you so that you had that urge to please someone.”

### Support

Many participants welcomed the help they received from health coaches and thought they were “very supportive.” Here is how one woman described her experience with the health coach:

“I liked the fact that I had someone on my team. She never said I was doing something wrong so it was good positive reinforcement. It was great to bounce stuff off...My coach helped me be able to better grasp the need to do these things and I think at this point, the fruit has become a habit that I will keep after the baby. Now I’m loving the fruit. [My husband] comes in with tangerines and now I’m tearing the fruit up!”

One participant described her preference for phone communication over texts or emails from health coaches, she noted that the phone calls allowed the health coach to show “she had a supportive nature and genuinely was concerned.” Another woman agreed that this personal touch was important: “I always thought she was like a friend and she would go above and beyond to find answers. We talked about certain goals. I felt like I could go to her even if it wasn’t for a call. If had a question I could reach out to her.”

### Wishes for more integration

When asked about how the intervention could be improved, some women wished the health coach interactions were “more closely tied with the doctor’s appointments… It was too removed from the medical side.” Another woman agreed that incorporating the intervention into the clinic would be more convenient: “more integration with the doctors in general. If you’re already going in to see the doc once a month it would be great to see someone [a health coach] there already.” Besides convenience, another participant explained that more integration would also help keep herself accountable: “If the health coach had access to medical records she could ask what has happened over the last two weeks if she sees you gained 2 pounds. Sometimes you don’t want to admit you gained weight to a health coach, so it would be better if she already knew.”

### Desire for more resources

Although the majority of participants thought the intervention was useful, several women said they “want more resources, not just a check in to talk.” Another participant explained that “all the goals are just discussed verbally. Maybe having more concrete goals that are maybe a worksheet or something.” One woman suggested “there’s no way to track your goal other than a conversation, so if there was some study app that made it a little more automated.” Another woman proposed front-loading these additional resources: “Maybe offering some guidelines in the beginning…starting off with ‘try these meal planners’ or ‘try this calorie tracking app.’”

## Discussion

In this pilot study, we found that a remote health coach intervention during pregnancy was feasible and that women reported high satisfaction. Surveys showed participants placed a high level of trust in healthcare providers, and 81% (21 of 26) said health coaches motivated them to achieve their goals. Although we did not find differences in food or beverage consumption frequency, fewer participants at follow-up than at baseline worried about being able in post-partum to lose the weight that they gained during pregnancy. In interviews, women primarily cited their own health, sometimes adding the health of the baby, as the main source of motivation to achieve lifestyle goals. Several women liked the supportive approach used by health coaches and most thought health coaches kept them accountable, particularly because coaches served as a third-party resource outside of family/friends and healthcare providers. Participants suggested more integration with clinical care and adding visual materials (e.g. goal worksheets or mobile apps) to improve future remote health coach interventions.

Previous lifestyle interventions have focused on adapting the approach and content (e.g. type of physical activity, diet) of the intervention to the individual [33]. We now see a need to individualize interventions according to participant technology preferences including methods of communication with health coaches, and goal tracking. For example, authors of one RCT designed to reduce behavioral cancer risk factors found that equal proportions of participants chose to receive intervention materials via print and the web [41], and concluded in another study that using just one modality of communication (text messages vs. automated voice responses) with participants may limit efficacy [42]. Although we considered implementing a study mobile app to track goals in this study, we decided against an app based on findings from focus groups prior to our pilot study that such an app would be “not that useful” [43]. However, in the current pilot study, several participants in interviews proposed adding more visual materials and tracking tools (both paper goal worksheets and mobile apps) in future interventions, suggesting that individuals have a wide range of preferences regarding how technology can facilitate behavior change.

In recent large RCTs of lifestyle interventions during pregnancy, just 19% of eligible women in the UPBEAT study and 40% in the LIMIT study decided to enroll. In contrast, while considering this was a pilot study in a selected population, 81% (30 out of 37) of potentially eligible and 100% (30 out of 30) of approached women enrolled in our pilot study. We hypothesize that the flexible modes of remote communication (phone, email, text) with health coaches allowed for a more individualized delivery of the intervention that improved enrollment compared to interventions proposed in UPBEAT and LIMIT trials that requested up to 1.5 h weekly face-to-face sessions [27, 28].

Our surveys support previous qualitative research that pregnant women put a lot of trust in advice about weight-related issues from clinicians [43], yet some (19%, 5 of 26) participants in end of pregnancy surveys reported that none of their healthcare providers (neither doctor nor midwife) discussed GWG with them. Our previous study found that healthcare providers often hesitated to spontaneously offer information about appropriate GWG or about how to make lifestyle changes to achieve appropriate weight gain [43], and other research on clinicians found that many of them are uncomfortable discussing weight with pregnant women [30, 44]. These reports along with our interview findings that the health coach’s position as a third-party resource was helpful, suggest that support from a health coach to adopt lifestyles encouraging appropriate GWG could be useful for both pregnant women and clinicians.

### Limitations

We conducted this study at one clinic with a high socioeconomic sample of women, so results may vary for other women depending on the clinic and location; however, the ethnicities and ages of study participants were similar to the overall group medical practice. Because this study was a one-arm pilot without a control group, we were unable to determine if differences between baseline and follow-up surveys were responses to the intervention or the results of a progressing pregnancy. A few participants in interviews expressed dissatisfaction with the intervention after some lag time occurred when switching a few participants’ health coach due to an unexpected event unrelated to the study. Future interventions could use multiple health coaches to avoid unanticipated break of continuity in health coach support. Another limitation of the study is that we did not measure GWG. Yet, based on electronic medical record data, we observed that 35.7% (10 of 28) had excessive GWG, which is less than the 62% of overweight women and 56% of women with obesity who had excessive GWG in the national report based on CDC data from 2012 to 2013 [3].

## Conclusions

In this remote health coach intervention pilot study, we found that we can recruit very effectively, that the intervention was acceptable, and that participants reported high satisfaction. Although it remains to be tested with a larger study population, the efficient recruitment, remote methods of intervention delivery, and modest research expenses suggest scalability of the intervention. Based on the relatively unsuccessful trials using lifestyle interventions during pregnancy, there is recent interest in targeting at-risk women prior to pregnancy. We feel that some of the lessons learned from this pilot study could be applied to behavioral studies with pregnant women or to pre-pregnancy designs [45, 46]. We propose that future interventions targeting women of reproductive age with excess weight include a supportive, integrated health coach intervention that includes the rest of the health care team to provide one coordinated front to educate and motivate patients. Future interventions should be personalized not only in the approach and content, but also to the women’s preferences in mode of communication and technological tools to support goals tracking.

## Additional file

Additional file 1: Details **Tables S1–S5.** (DOCX 35 kb)

## Abbreviation

GWG: Gestational weight gain

## References

[1] Flegal KM, Carroll MD, Kit BK, Ogden CL (2012). Prevalence of obesity and trends in the distribution of body mass index among US adults, 1999-2010. JAMA.

[2] Deputy NP, Sharma AJ, Kim SY, Hinkle SN (2015). Prevalence and characteristics associated with gestational weight gain adequacy. Obstet Gynecol.

[3] Deputy NP, Sharma AJ, Kim SY (2015). Gestational weight gain - United States, 2012 and 2013. MMWR Morb Mortal Wkly Rep.

[4] Nehring I, Schmoll S, Beyerlein A, Hauner H, von Kries R (2011). Gestational weight gain and long-term postpartum weight retention: a meta-analysis. Am J Clin Nutr.

[5] Oken E, Kleinman KP, Belfort MB, Hammitt JK, Gillman MW (2009). Associations of gestational weight gain with short- and longer-term maternal and child health outcomes. Am J Epidemiol.

[6] Walter JR, Perng W, Kleinman KP, Rifas-Shiman SL, Rich-Edwards JW, Oken E (2015). Associations of trimester-specific gestational weight gain with maternal adiposity and systolic blood pressure at 3 and 7 years postpartum. Am J Obstet Gynecol.

[7] Viswanathan M, Siega-Riz AM, Moos MK, Deierlein A, Mumford S, Knaack J, Thieda P, Lux LJ, Lohr KN (2008). Outcomes of maternal weight gain. Evid Rep Technol Assess (Full Rep).

[8] Fraser A, Tilling K, Macdonald-Wallis C, Sattar N, Brion MJ, Benfield L, Ness A, Deanfield J, Hingorani A, Nelson SM (2010). Association of maternal weight gain in pregnancy with offspring obesity and metabolic and vascular traits in childhood. Circulation.

[9] Hivert MF, Rifas-Shiman SL, Gillman MW, Oken E (2016). Greater early and mid-pregnancy gestational weight gains are associated with excess adiposity in mid-childhood. Obesity (Silver Spring).

[10] Hochner H, Friedlander Y, Calderon-Margalit R, Meiner V, Sagy Y, Avgil-Tsadok M, Burger A, Savitsky B, Siscovick DS, Manor O (2012). Associations of maternal prepregnancy body mass index and gestational weight gain with adult offspring cardiometabolic risk factors: the Jerusalem perinatal family follow-up study. Circulation.

[11] Karachaliou M, Georgiou V, Roumeliotaki T, Chalkiadaki G, Daraki V, Koinaki S, Dermitzaki E, Sarri K, Vassilaki M, Kogevinas M (2015). Association of trimester-specific gestational weight gain with fetal growth, offspring obesity, and cardiometabolic traits in early childhood. Am J Obstet Gynecol.

[12] Oken E, Rifas-Shiman SL, Field AE, Frazier AL, Gillman MW (2008). Maternal gestational weight gain and offspring weight in adolescence. Obstet Gynecol.

[13] Oken E, Taveras EM, Kleinman KP, Rich-Edwards JW, Gillman MW (2007). Gestational weight gain and child adiposity at age 3 years. Am J Obstet Gynecol.

[14] Parker M, Rifas-Shiman SL, Oken E, Belfort MB, Jaddoe VW, Gillman MW (2012). Second trimester estimated fetal weight and fetal weight gain predict childhood obesity. J Pediatr.

[15] Bogaerts A F L, Devlieger R, Nuyts E, Witters I, Gyselaers W, Van den Bergh B R H (2012). Effects of lifestyle intervention in obese pregnant women on gestational weight gain and mental health: a randomized controlled trial. International Journal of Obesity.

[16] Harrison CL, Lombard CB, Strauss BJ, Teede HJ (2013). Optimizing healthy gestational weight gain in women at high risk of gestational diabetes: a randomized controlled trial. Obesity (Silver Spring).

[17] Herring SJ, Cruice JF, Bennett GG, Rose MZ, Davey A, Foster GD (2016). Preventing excessive gestational weight gain among African American women: a randomized clinical trial. Obesity (Silver Spring).

[18] Huang TT, Yeh CY, Tsai YC (2011). A diet and physical activity intervention for preventing weight retention among Taiwanese childbearing women: a randomised controlled trial. Midwifery.

[19] Renault KM, Norgaard K, Nilas L, Carlsen EM, Cortes D, Pryds O, Secher NJ (2014). The Treatment of Obese Pregnant Women (TOP) study: a randomized controlled trial of the effect of physical activity intervention assessed by pedometer with or without dietary intervention in obese pregnant women. Am J Obstet Gynecol.

[20] Vesco KK, Karanja N, King JC, Gillman MW, Leo MC, Perrin N, McEvoy CT, Eckhardt CL, Smith KS, Stevens VJ (2014). Efficacy of a group-based dietary intervention for limiting gestational weight gain among obese women: a randomized trial. Obesity (Silver Spring).

[21] Vesco KK, Karanja N, King JC, Gillman MW, Leo MC, Perrin N, McEvoy CT, Eckhardt CL, Smith KS, Stevens VJ (2014). Efficacy of a group-based dietary intervention for limiting gestational weight gain among obese women: a randomized trial. Obesity.

[22] Guelinckx I, Devlieger R, Mullie P, Vansant G (2010). Effect of lifestyle intervention on dietary habits, physical activity, and gestational weight gain in obese pregnant women: a randomized controlled trial. Am J Clin Nutr.

[23] Hawkins M, Hosker M, Marcus BH, Rosal MC, Braun B, Stanek EJ, Markenson G, Chasan-Taber L (2015). A pregnancy lifestyle intervention to prevent gestational diabetes risk factors in overweight Hispanic women: a feasibility randomized controlled trial. Diabet Med.

[24] Hui A, Back L, Ludwig S, Gardiner P, Sevenhuysen G, Dean H, Sellers E, McGavock J, Morris M, Bruce S (2012). Lifestyle intervention on diet and exercise reduced excessive gestational weight gain in pregnant women under a randomised controlled trial. BJOG.

[25] Phelan S, Phipps MG, Abrams B, Darroch F, Grantham K, Schaffner A, Wing RR (2014). Does behavioral intervention in pregnancy reduce postpartum weight retention? Twelve-month outcomes of the fit for delivery randomized trial. Am J Clin Nutr.

[26] Skouteris H, McPhie S, Hill B, McCabe M, Milgrom J, Kent B, Bruce L, Herring S, Gale J, Mihalopoulos C (2016). Health coaching to prevent excessive gestational weight gain: a randomized-controlled trial. Br J Health Psychol.

[27] Dodd JM, Turnbull D, McPhee AJ, Deussen AR, Grivell RM, Yelland LN, Crowther CA, Wittert G, Owens JA, Robinson JS (2014). Antenatal lifestyle advice for women who are overweight or obese: LIMIT randomised trial. BMJ.

[28] Poston L, Bell R, Croker H, Flynn AC, Godfrey KM, Goff L, Hayes L, Khazaezadeh N, Nelson SM, Oteng-Ntim E (2015). Effect of a behavioural intervention in obese pregnant women (the UPBEAT study): a multicentre, randomised controlled trial. Lancet Diabetes Endocrinol.

[29] Brown MJ, Sinclair M, Liddle D, Hill AJ, Madden E, Stockdale J (2012). A systematic review investigating healthy lifestyle interventions incorporating goal setting strategies for preventing excess gestational weight gain. PLoS One.

[30] Campbell F, Johnson M, Messina J, Guillaume L, Goyder E (2011). Behavioural interventions for weight management in pregnancy: a systematic review of quantitative and qualitative data. BMC Public Health.

[31] Gardner B, Wardle J, Poston L, Croker H (2011). Changing diet and physical activity to reduce gestational weight gain: a meta-analysis. Obes Rev.

[32] Streuling Ina, Beyerlein Andreas, von Kries Rüdiger (2010). Can gestational weight gain be modified by increasing physical activity and diet counseling? A meta-analysis of interventional trials. The American Journal of Clinical Nutrition.

[33] Flynn AC, Dalrymple K, Barr S, Poston L, Goff LM, Rogozinska E, van Poppel MN, Rayanagoudar G, Yeo S, Barakat Carballo R (2016). Dietary interventions in overweight and obese pregnant women: a systematic review of the content, delivery, and outcomes of randomized controlled trials. Nutr Rev.

[34] Taveras EM, Gortmaker SL, Hohman KH, Horan CM, Kleinman KP, Mitchell K, Price S, Prosser LA, Rifas-Shiman SL, Gillman MW (2011). Randomized controlled trial to improve primary care to prevent and manage childhood obesity: the high five for kids study. Arch Pediatr Adolesc Med.

[35] Hettema J, Steele J, Miller WR (2005). Motivational interviewing. Annu Rev Clin Psychol.

[36] Wurtele SK, Maddux JE (1987). Relative contributions of protection motivation theory components in predicting exercise intentions and behavior. Health Psychol.

[37] Janz Nancy K., Becker Marshall H. (1984). The Health Belief Model: A Decade Later. Health Education Quarterly.

[38] Bandura A (2004). Health promotion by social cognitive means. Health Educ Behav.

[39] Sacco RL (2011). The new American Heart Association 2020 goal: achieving ideal cardiovascular health. J Cardiovasc Med (Hagerstown).

[40] Crabtree BF, Miller WL (1999). Doing qualitative research.

[41] Greaney ML, Puleo E, Bennett GG, Haines J, Viswanath K, Gillman MW, Sprunck-Harrild K, Coeling M, Rusinak D, Emmons KM (2014). Factors associated with choice of web or print intervention materials in the healthy directions 2 study. Health Educ Behav.

[42] Greaney ML, Puleo E, Sprunck-Harrild K, Bennett GG, Cunningham MA, Gillman MW, Coeling M, Emmons KM (2012). Electronic reminders for cancer prevention: factors associated with preference for automated voice reminders or text messages. Prev Med.

[43] Criss S, Oken E, Guthrie L, Hivert MF (2016). A qualitative study of gestational weight gain goal setting. BMC Pregnancy Childbirth.

[44] Oken E, Switkowski K, Price S, Guthrie L, Taveras EM, Gillman M, Friedes J, Callaghan W, Dietz P (2013). A qualitative study of gestational weight gain counseling and tracking. Matern Child Health J.

[45] Catalano P, deMouzon SH (2015). Maternal obesity and metabolic risk to the offspring: why lifestyle interventions may have not achieved the desired outcomes. Int J Obes.

[46] Rönö K, Stach-Lempinen B, Klemetti MM, Kaaja RJ, Pöyhönen-Alho M, Eriksson JG, Koivusalo SB (2014). Prevention of gestational diabetes through lifestyle intervention: study design and methods of a Finnish randomized controlled multicenter trial (RADIEL). BMC Pregnancy and Childbirth.

