# Supplementary material for: Supporting healthful lifestyles during pregnancy: a health coach intervention pilot study
Source: BMC Pregnancy Childbirth. 2018 Sep 17;18:375. doi: 10.1186/s12884-018-2010-z (PMC6142676; doi:10.1186/s12884-018-2010-z)
Supplement: Supplementary file 1 — Details Tables S1–S5. (DOCX 35 kb) [file 12884_2018_2010_MOESM1_ESM.docx]

**Supporting Healthful Lifestyles during Pregnancy: A Health Coach Intervention Pilot Study – Supplemental Materials**

Supp Table 1: Interview guide used for follow-up phone interviews.

| \|  \| **Domain** \| **Question** \| \| --- \| --- \| --- \| \| 1 \| Goals \| What health goals did you set for yourself during this pregnancy? \| \| 2 \| Goals \| What helped you achieve your goals? \| \| 3 \| Motivation \| What motivated you to achieve your goals? \| \| 4 \| Health Coach \| How would you describe your experience with the health coach? \| \| 5 \| Communication \| What did you think about the different components for delivery of the intervention including face-to-face meetings, phone conversations, texts, or emails? \| \| 6 \| Communication \| How often were you contacted by phone? By email? By text? Was that frequency adequate? \| \| 7 \| Improvements \| How would you improve the intervention? \| |
| --- | --- | --- | --- | --- | --- | --- | --- | --- | --- | --- | --- | --- | --- | --- | --- | --- | --- | --- | --- | --- | --- | --- | --- | --- |

Supp Table 2: List of goals proposed to participants and optimal levels to be reached.

| **Domain** | **Targeted behaviors (menu)** | **Optimal intervention goals** |
| --- | --- | --- |
| Diet | Increase vegetables and fruits intake | Vegetables + fruits >4.5 cups/day * |
|  | Increase whole grains intake | Whole grains >3 servings/day * |
|  | Increase low mercury (Hg) fish intake | Low-Hg fish intake =2 servings/week * |
|  | Decrease fast-food intake | Fast-food <1 time/ week * |
|  | Decrease sugar-sweetened beverages (SSB) intake | No SSB * |
| Physical activity | Increase number of steps/day | Walk > 10,000 steps/day |
|  | Increase moderate activity | Moderate activity ≥ 5 times/week or 150min/week* |
| Screen time | Decrease screen time | TV + other < 2h/day |
| Sleep time | Optimize sleep duration | 6 to 8h/day |

*These lifestyle targets are in line with the Life’s Simple 7 health factors from the American Heart Association.

Supp Table 3: Beverage consumption frequency from the prior month in surveys at baseline and at the end of the intervention.

|  | **Baseline** | | | | | | **End of Intervention** | | | | | |  |
| --- | --- | --- | --- | --- | --- | --- | --- | --- | --- | --- | --- | --- | --- |
| **Beverage** | **N** | **Less than once per week N (%)** | **Once per week N (%)** | **2-4 times per week N (%)** | **Nearly daily or daily N (%)** | **Twice or more per day N (%)** | **N** | **Less than once per week N (%)** | **Once per week N (%)** | **2-4 times per week N (%)** | **Nearly daily or daily N (%)** | **Twice or more per day N (%)** | **P-value** |
| **100% fruit juice** | 30 | 9 (30) | 4 (13) | 8 (27) | 8 (27) | 1 (3) | 26 | 7 (27) | 3 (12) | 10 (38) | 6 (23) | 0 (0) | 0.76 |
| **Soda with sugar (Coke, Sprite, Pepsi)** | 30 | 19 (63) | 5 (17) | 4 (13) | 2 (7) | 0 (0) | 26 | 18 (69) | 5 (19) | 2 (8) | 1 (4) | 0 (0) | 0.63 |
| **Other drink with sugar (sweet tea, fruit punch)** | 30 | 17 (57) | 5 (17) | 6 (20) | 1 (3) | 1 (3) | 26 | 18 (69) | 3 (12) | 5 (19) | 0 (0) | 0 (0) | 0.80 |
| **Diet Soda (Diet Coke, Diet Sprite)** | 30 | 26 (87) | 3 (10) | 0 (0) | 1 (3) | 0 (0) | 25 | 24 (96) | 1 (4) | 0 (0) | 0 (0) | 0 (0) | 0.57 |
| **Other flavored drink without sugar (Crystal Light, sugar-free iced tea)** | 30 | 25 (83) | 0 (0) | 3 (10) | 0 (0) | 2 (7) | 25 | 20 (80) | 2 (8) | 3 (12) | 0 (0) | 0 (0) | 0.53 |

**P*-value calculated from pre- and post-intervention surveys using the Bowker’s Test of Symmetry.

Supp Table 4: Food consumption frequency from the prior month in surveys at baseline and at the end of the intervention.

|  | **Baseline** | | | | | | **End of Intervention** | | | | | |  |
| --- | --- | --- | --- | --- | --- | --- | --- | --- | --- | --- | --- | --- | --- |
| **Food Item** | **N** | **Less than once per week N (%)** | **Once per week N (%)** | **2-4 times per week N (%)** | **Nearly daily or daily N (%)** | **Twice or more per day N (%)** | **N** | **Less than once per week N (%)** | **Once per week N (%)** | **2-4 times per week N (%)** | **Nearly daily or daily N (%)** | **Twice or more per day N (%)** | **P-value** |
| **whole milk dairy foods (whole milk, hard cheese, butter, ice cream)** | 30 | 6 (20) | 5 (17) | 13 (43) | 5 (17) | 1 (3) | 26 | 8 (31) | 3 (12) | 11 (42) | 2 (8) | 2 (8) | 0.71 |
| **low-fat milk products (skim milk, low-fat yogurt, low-fat cottage cheese)** | 30 | 7 (23) | 1 (3) | 12 (40) | 8 (27) | 2 (7) | 25 | 3 (12) | 2 (8) | 7 (28) | 11 (44) | 2 (8) | 0.90 |
| **whole grain foods (whole grain bread, brown rice, cereal)** | 30 | 1 (3) | 6 (20) | 11 (37) | 10 (33) | 2 (7) | 26 | 2 (8) | 0 (0) | 8 (31) | 14 (54) | 2 (8) | 0.63 |
| **pasta, rice, or noodles** | 30 | 2 (7) | 6 (20) | 18 (60) | 4 (13) | 0 (0) | 26 | 5 (19) | 7 (27) | 10 (38) | 4 (15) | 0 (0) | 0.55 |
| **baked products (donuts, cookies, muffins, cake, crackers)** | 30 | 9 (30) | 12 (40) | 7 (23) | 2 (7) | 0 (0) | 26 | 6 (23) | 5 (19) | 12 (46) | 3 (12) | 0 (0) | 0.20 |
| **deep fried foods (fried chicken or fish, french fries, onion rings)** | 30 | 14 (47) | 9 (30) | 7 (23) | 0 (0) | 0 (0) | 26 | 20 (77) | 4 (15) | 2 (8) | 0 (0) | 0 (0) | 0.08 |
| **vegetables (fresh, frozen, or canned)** | 30 | 0 (0) | 3 (10) | 10 (33) | 12 (40) | 5 (17) | 26 | 0 (0) | 3 (12) | 4 (15) | 12 (46) | 7 (27) | 0.54 |
| **fruit** | 30 | 1 (3) | 3 (10) | 11 (37) | 7 (23) | 8 (27) | 26 | 2 (8) | 2 (8) | 4 (15) | 8 (31) | 10 (38) | 0.36 |
| **fish** | 29 | 14 (48) | 11 (38) | 4 (14) | 0 (0) | 0 (0) | 25 | 13 (52) | 10 (40) | 2 (8) | 0 (0) | 0 (0) | 0.64 |

**P*-value calculated from pre- and post-intervention surveys using the Bowker’s Test of Symmetry.

Supp Table 5: Fast-food consumption frequency from the prior month at baseline and at the end of the intervention.

| **Baseline** | | | | | | | **End of Intervention** | | | | | | |  |
| --- | --- | --- | --- | --- | --- | --- | --- | --- | --- | --- | --- | --- | --- | --- |
| **N** | **Never N (%)** | **1-3 times in the past month N (%)** | **1 or 2 times per week N (%)** | **3 or 4 times per week N (%)** | **5 or 6 times per week N (%)** | **7 or more times per week N (%)** | **N** | **Never N (%)** | **1-3 times in the past month N (%)** | **1 or 2 times per week N (%)** | **3 or 4 times per week N (%)** | **5 or 6 times per week N (%)** | **7 or more times per week N (%)** | **P-value** |
| 30 | 11 (37) | 13 (43) | 4 (13) | 2 (7) | 0 (0) | 0 (0) | 26 | 8 (31) | 16 (62) | 2 (8) | 0 (0) | 0 (0) | 0 (0) | 0.89 |

**P*-value calculated from pre- and post-intervention surveys using the Bowker’s Test of Symmetry
